# Sex-differences in proteasome-dependent K48-polyubiquitin signaling in the amygdala are developmentally regulated in rats

**DOI:** 10.1186/s13293-023-00566-z

**Published:** 2023-11-10

**Authors:** Kayla Farrell, Aubrey Auerbach, Catherine Liu, Kiley Martin, Myasia Pareno, W. Keith Ray, Richard F. Helm, Fernando Biase, Timothy J. Jarome

**Affiliations:** 1 School of Animal Sciences, Virginia Polytechnic Institute and State University, 175 West Campus Dr., 2150 Litton-Reaves Hall, Blacksburg, VA 24061 USA; 2 Department of Biological Sciences, Virginia Polytechnic Institute and State University, Blacksburg, VA USA; 3 School of Neuroscience, Virginia Polytechnic Institute and State University, 175 West Campus Dr., 2150 Litton-Reaves Hall, Blacksburg, VA 24061 USA; 4 Department of Biochemistry, Virginia Polytechnic Institute and State University, Blacksburg, VA USA

**Keywords:** Ubiquitin, Sex differences, Amygdala, Memory, Baseline

## Abstract

**Background:**

Sex differences have been observed in several brain regions for the molecular mechanisms involved in baseline (resting) and memory-related processes. The ubiquitin proteasome system (UPS) is a major protein degradation pathway in cells. Sex differences have been observed in lysine-48 (K48)-polyubiquitination, the canonical degradation mark of the UPS, both at baseline and during fear memory formation within the amygdala. Here, we investigated when, how, and why these baseline sex differences arise and whether both sexes require the K48-polyubiquitin mark for memory formation in the amygdala.

**Methods:**

We used a combination of molecular, biochemical and proteomic approaches to examine global and protein-specific K48-polyubiquitination and DNA methylation levels at a major ubiquitin coding gene (*Uba52*) at baseline in the amygdala of male and female rats before and after puberty to determine if sex differences were developmentally regulated. We then used behavioral and genetic approaches to test the necessity of K48-polyubiquitination in the amygdala for fear memory formation.

**Results:**

We observed developmentally regulated baseline differences in *Uba52* methylation and total K48-polyubiquitination, with sexual maturity altering levels specifically in female rats. K48-polyubiquitination at specific proteins changed across development in both male and female rats, but sex differences were present regardless of age. Lastly, we found that genetic inhibition of K48-polyubiquitination in the amygdala of female, but not male, rats impaired fear memory formation.

**Conclusions:**

These results suggest that K48-polyubiquitination differentially targets proteins in the amygdala in a sex-specific manner regardless of age. However, sexual maturity is important in the developmental regulation of K48-polyubiquitination levels in female rats. Consistent with these data, K48-polyubiquitin signaling in the amygdala is selectively required to form fear memories in female rats. Together, these data indicate that sex-differences in baseline K48-polyubiquitination within the amygdala are developmentally regulated, which could have important implications for better understanding sex-differences in molecular mechanisms involved in processes relevant to anxiety-related disorders such as post-traumatic stress disorder (PTSD).

**Supplementary Information:**

The online version contains supplementary material available at 10.1186/s13293-023-00566-z.

## Plain language summary

Male and female brains have differences in size, development, and cellular processes. Further, males and females have differences in likelihood of developing certain anxiety-related disorders, such as post-traumatic stress disorder (PTSD). We previously observed sex differences in a cellular mechanism that controls the destruction of proteins via tagging by the protein modifier ubiquitin in resting and behaviorally trained animals. We found that adult female rats “ubiquitinated” different proteins during learning and had more ubiquitin than male rats at rest in the amygdala, the brain region that controls emotional regulation. This study investigated if the sex difference in ubiquitin at rest changed as animals age, including the proteins being ubiquitinated and how the amount of ubiquitin was controlled. We also investigated if male and female rats need ubiquitin for memory formation. We found that males and females ubiquitinate different proteins, but that aging also contributes to changes in this, suggesting that sexual maturity may be important for controlling the amount of ubiquitin in females. Lastly, we found that only female rats needed ubiquitin in the amygdala for forming a fear memory. These results are important for understanding the role of ubiquitin activity at different developmental stages and for forming fear-based memories in both sexes. Since females are more likely to develop PTSD than males, these data could help understand how different cellular processes work together in PTSD development to create better treatment options.

**Supplementary Information:**

The online version contains supplementary material available at 10.1186/s13293-023-00566-z.

## Highlights

- K48-polyubiquitination, the canonical protein degradation mark, differentially targets proteins at baseline in the amygdala of male and female rats across development.
- DNA methylation at *Uba52,* a major ubiquitin coding gene, and overall K48-polyubiquitination levels are developmentally regulated in female rats.
- Female, but not male, rats require K48-polyubiquitin signaling in the amygdala for fear memory formation, suggesting males may utilize another linkage for protein degradation processes during fear memory formation.

**Supplementary Information:**

The online version contains supplementary material available at 10.1186/s13293-023-00566-z.

## Background

It is difficult to decipher how males and females truly differ in underlying biological mechanisms, as most research has been conducted only in males, but the inclusion of females during experimental design has led to the discovery of a wide array of sex-differences in mechanisms involved in areas of study ranging from the skeletal muscle [1, 2] to adipose tissue [3, 4] and the brain [5–10]. Within the brain, sex differences have been observed in different regions [10–14] as well as in memory [15–21] and prevalence of psychological disorders [22–24]. For example, in humans, females are 2–3 times more likely to develop post-traumatic stress disorder (PTSD) than males, despite not experiencing more traumatic events [22], though the reasons for this sex bias remain unknown. Studies interested in sex biases in the development of specific psychiatric and anxiety disorders have focused efforts on distinguishing sex differences in specific brain regions, such as the amygdala, which is a center for emotional processing in the brain. Sex-differences have been observed within the amygdala in terms of size and proportion of subnuclei across age, which are functionally distinct [13]. Another study observed sex-differences in structural maturation of both the amygdala and hippocampus, which is critically involved in memory formation. Sex differences were observed in the growth rate of the human amygdala, with rapid deceleration of growth occurring around 13 years in females, but not until 20 years in males [25]. Interestingly, when looking at amygdala response to familiar negative images in humans, it has been observed that adult females show a sustained amygdala response while males do not [26]. In terms of memory, it has been observed that females out-perform males in verbal memory and recall of faces and odors, while males out-perform females in spatial memory [27]. Fascinatingly, a rodent study observed sex differences in spatial memory were regulated by puberty, where prepubescent females out-perform prepubescent males, but adult males out-perform adult females in an object location memory task [28]. Another rodent study identified sex-specific developmental patterns in context fear expression across adolescence to adulthood, where male rats showed increasing, and female rats showed decreasing, context fear expression across development [29]. These studies allude to the presence of sex differences within the brain at baseline and during memory processes that may be developmentally regulated, though, to date, the molecular mechanisms underlying these differences has not been extensively studied.

The ubiquitin–proteasome system (UPS) is a highly conserved pathway that controls the majority of protein degradation in cells at baseline and is involved in memory formation [30–32]. The UPS labels proteins of interest by covalently attaching either one (monoubiquitination) or multiple (polyubiquitination) ubiquitin proteins, with the latter using one of its eight linkage sites on the previous ubiquitin. Each ubiquitin linkage site has a unique role in the cell, with some leading to protein degradation by the proteasome complex and others having non-proteolytic functions. Interestingly, sex-differences in UPS signaling have been observed at baseline [18] and during fear memory formation [15, 18–20, 33]. At baseline, we found that 9-week-old adult female rats have more overall ubiquitin in the amygdala compared to 9-week-old adult males, which correlated with higher 5-hydroxymethylcytosine levels, a DNA methylation mark typically associated with transcriptional activation, at the promoter of *Uba52,* one of the four ubiquitin coding genes [18]. During fear memory formation, we previously discovered sex-specific protein targets of lysine-48 (K48) polyubiquitination, the highly abundant, canonical protein degradation mark, in the amygdala of 9-week-old adult male and female rats [19]. Another study looking at trace fear conditioning observed differences in K48-polyubiquitination in the amygdala of aged (2 year old) male, but not female rats [15]. Together, these data suggest K48-polyubiquitination has sex-specific differential expression and function in the amygdala, but it is unclear whether sex-differences in baseline expression observed in adults is developmentally regulated and if K48-polyubiquitin signaling is necessary for fear memory formation in both sexes.

Here, we identified protein targets of K48-polyubiquitination in the amygdala of 4- (prepubescent) and 9-week-old (sexually mature) male and female rats at baseline through liquid chromatography mass spectroscopy (LC/MS) to determine if K48-polyubiquitin signaling is developmentally regulated in a sex-specific manner. We then determined the methylation pattern at the *Uba52* promoter through direct bisulfite sequencing and quantified total K48-polyubiquitination levels in the amygdala of the same animals to better understand the relationship between DNA methylation, total protein levels, and targeting of protein substrates across development. Lastly, we used the CRISPR-dCas13b-ADAR2DD system to knockdown the ubiquitin K48 linkage in the amygdala of 9-week-old adult male and female rats, trained rats to a contextual fear paradigm, and then tested memory retention to determine whether K48-polyubiquitin signaling is necessary for fear memory formation in both sexes. Together, these data provide a deeper understanding of baseline and memory-specific sex differences in ubiquitin signaling within the amygdala, expanding the knowledge of potential mechanisms contributing to sex differences in the prevalence of amygdala-dependent disorders, such as PTSD.

## Methods

### Subjects

Ten male and 10 female 4-week-old, and 34 male and 26 female 8- to 9-week-old Sprague Dawley rats were used. All animals were obtained from Envigo (Fredrick, MA) and upon arrival were housed two per cage with free access to water and rat chow. Male and female rats were housed in the same room using ventilated racks and, in all cases, males and females were ran simultaneously for behavioral and molecular assays. Likewise, all male and female subjects were shipped at the same age and arrived on the same date. For experiments using 4-week-old and 9-week-old animals, rats were shipped at either 3 weeks or 8 weeks of age, respectively, allowed 48 hours to recover from shipping, then handled four consecutive days before being euthanized on the seventh day post-arrival. Animals were maintained under a 12:12-h light/dark cycle, with experiments and animal handling taking place only during the light portion of the cycle. All animals were handled for 4 days prior to behavioral experiments or euthanasia. Animals within a cage were randomly assigned to experimental groups and while researchers were not blinded to group allocation, they were unable to distinguish between animals during the behavioral procedures. All procedures were approved by the Virginia Polytechnic Institute and State University Institutional Animal Care and Use Committee (protocols #20-233) and conducted with the ethical guidelines of the National Institutes of Health.

### Plasmid cloning

The CMV-dPspCas13b-GS-ADAR2DD(E488Q/T375G)-delta-984-1090 (Addgene #103871) plasmid was used. The K48 guide RNA (gRNA) plasmid was generated using the pC0043-PspCas13b crRNA backbone (Addgene #103854) plasmid and validated in our prior study, where detailed methods and gRNA information can be found [20].

### Cranial infusion of plasmids

CRISPR plasmids were infused into the BLA as previously described [18, 20]. During surgery, animals were anesthetized with 1.5–4% isoflurane in 100% O_2_. Plasmids were bilaterally infused into the BLA using In Vivo Jet-PEI (Polyplus, Berkley, CA) as a transfection reagent, as previously described [18, 20]. Infusion of plasmids was done with a linear actuator set at a constant rate of 0.1 µl per minute for a total volume of 0.5 µl per side with the following coordinates related to bregma: AP -3.0 mm, ML ± 5.0 mm, DV -7.7 mm. Following surgery, animals recovered for at least 14 days before being handled. Behavioral procedures did not take place until 28 days after surgery.

### Behavioral apparatus

The two identical Habitest chambers used for contextual fear conditioning have been previously described by our group [19, 20]. Briefly, each chamber comprises a steel test cage with front and back Plexiglass walls, a grid shock floor, and plastic drop pan. In the top back corner of the chamber is a house light, which remained on during behavioral procedures. Each chamber sits within an isolation cubicle that contains an acoustic liner and a house fan, which remained active during behavioral procedures Behavior was recorded and stored for later analysis using a USB camera mounted at a 45° angle outside of the back Plexiglass wall of the chamber. A precision animal shocker under the control of FreezeFrame 4 software was used to deliver a shock through the grid floor during training. FreezeFrame 4 software was also used to analyze animal behavior in real-time, using a freezing threshold of 2.0. The chamber walls were wiped with 70% isopropanol before use and between animals.

### Behavioral procedures

Behavioral procedures have been previously outlined [20]. Briefly, animals were handled 4 days prior to contextual fear conditioning. During the training session, animals were placed into the fear conditioning apparatus, given a 1 min baseline, and then received 4 unsignaled footshock (1.0 mA, 1 s, 59 s ITI) presentations, followed by a 1 min post-shock period. Animals were then placed back into their homecages. One day later, animals were placed back into the fear conditioning apparatus for 5 min, during which time no footshock presentations were given, and then returned to their homecage.

### Tissue collection

Rats were placed in a necrosis chamber and overdosed on isoflurane. Animals were decapitated and the brain was immediately removed and frozen on dry ice. Animals for baseline experiments were euthanized in the morning hours. Animals that underwent training were euthanized one day after the testing session. Both hemispheres of the BLA and the CA1 region of the hippocampus were then dissected out by blocking the brain in a rat brain matrix (Harvard Apparatus, Holliston, MA) incubated with dry ice. All dissected tissue was frozen at -80 °C until needed.

### Whole cell protein extraction

BLA and CA1 tissue was homogenized in whole cell lysis buffer as previously described [19]. Briefly, one hemisphere of tissue was homogenized in 500 µl buffer and then transferred to a microcentrifuge tube stored on ice. Homogenates were centrifuged at 10,000 × g for 10 min at 4 °C, the supernatant was collected, and then the Bio-Rad DC protein assay was used to measure protein concentration.

### Tandem ubiquitin binding entity

Tandem ubiquitin binding entity (TUBE) for K48 purification was conducted using previously described procedures [19]. Briefly, the high affinity K48-specific TUBE (#UM407M, Life Sensors, Malvern, PA) was washed in wash buffer before whole cell BLA protein samples were added. The bead/protein mix was incubated on an end-over-end rotator for 2 h at 4 °C. Samples were washed, collected by incubating at 96 °C for 5 min at 800 rpm in 1X sample buffer (Bio-rad, Hercules, CA), and then cooled to room temperature. Supernatant was stored at − 80 °C for mass spectrometry or western blot.

### Liquid chromatography/mass spectrometry

Liquid chromatography mass spectrometry (LC/MS) was competed using our previously described detailed methods [19]. The mass spectrometry proteomics data have been deposited to the ProteomeXchange Consortium via the PRIDE [1] partner repository with the dataset identifier PXD042021 and 10.6019/PXD042021.

### DNA isolation

DNA was isolated from BLA tissue using the Qiagen Allprep kit (Germantown, MD) following manufacturer’s instruction. DNA concentration was measured on the Take3 (BioTek, Winooski, VT), normalized (50 ng), and used for direct bisulfite sequencing.

### Direct bisulfite sequencing

Direct bisulfite sequencing of the *Uba52* promoter using previously described methods [18]. Briefly, genomic DNA (50 ng) was bisulfite converted using the Qiagen Bisulfite Kit before the *Uba52* promoter was amplified with a semi-nested PCR protocol. HotStarTaq Master Mix (#203443, Qiagen) was used for PCR procedures. The following methyl-specific primers for were used: *Uba52* F1: TAAATAAAAATTATGTTTGAAAGGTAATAT; *Uba52* F2: AATAAAAATT.

ATGTTTGAAAGGTAATATAG; *Uba52* R: AAAAAAAACCAAACTATCTAAAACC. PCR products from the second PCR were purified using ExoSAP-IT (Affymetrix, Santa Clara, CA) and then sequenced with the reverse primers by the Genomics Sequencing Center at the Fralin Life Sciences Institute of Virginia Tech. Percent methylation of the CpG sites was determined by the ratio between peak values of guanine (G) and cytosine (C) measured using Chromas software, *C*/(*C* + *T*) * 100. Sample size was chosen for a large effect size based on prior work using similar methods [18, 34, 35].

### Western blot

Western blot was conducted using previously outlined procedures [19]. Briefly, normalized whole cell protein samples (10 µg) were ran through SDS-PAGE using 7% Acrylamide gels and then transferred onto a membrane using a Turbo Transfer System (Biorad). Membranes were incubated for 1 h at room temperature in a 50:50 blocking buffer (50% Licor TBS blocking buffer and 50% TBS + 0.1% Tween-20) and then incubated overnight at 4 °C in primary antibody in 50:50 blocking buffer. The next morning, membranes were washed with TBS + 0.1% Tween-20 (TBSt) and incubated for 45 min at room temperature in secondary antibody (1:40,000; goat anti-rabbit 700CW) in 50:50 blocking buffer. Membranes were then washed twice with TBSt and then rinsed with TBS before being imaged using the Odyssey Fc (LI-COR, Lincoln, NE). Image Studio Ver 5.2. was used to analyze visualized proteins. Membranes were stripped with 0.2 M NaOH followed by two TBSt washes to remove the primary antibody and then incubated for 1 h at room temperature in blocking buffer. Samples were normalized to β-actin, which was used as a loading control. OD levels were normalized to 4-week-old male rats in experiments with 4-week-old and 9-week-old animals or to 9-week-old male rats in experiments with only 9-week-old animals due to variability between gels requiring normalization [18, 36].

### Antibodies

Antibodies used for western blotting included K48 polyubiquitin (1:1000; #ab140601, Abcam, Cambridge, MA) and β-actin (1:1000; #4967, Cell Signaling, Danvers, MA).

### Statistical analysis

All data are presented as mean with standard error, with individual sample values included in bar graphs (not in line graphs). All group sizes, number of replications, and statistical tests are reported in the figure legends. Outliers were defined as data points falling two or more standard deviations from the group mean. One outlier was removed from 4-week-old female amygdala K48 polyubiquitination level western blot data and 4-week-old and 9-week-old female hippocampus K48 polyubiquitination level western blot data. Prism software (GraphPadSoftware, La Jolla, CA) was used to assess normality and analyze data. Training data were analyzed using three-way analysis of variance (AVOVA). All other data were analyzed using two-way ANOVA and Tukey’s honest significant difference post hoc test or two-tailed t-tests as indicated in figure legends. Nonparametric data were analyzed with Mann–Whitney U test.

LC/MS data were analyzed in a similar manner to previously described methods [37]. Data were first log transformed (Log_2_(x + 1)) and proteins identified as having more than 15 zero values were excluded from analysis. The transformed data were analyzed using a generalized mixed model (two-way ANOVA with Tukey’s honest significant difference post hoc test) with the R package ‘lme4’ to compare Age, Sex and an interaction between Age X Sex, with subject as a random effect. Two-tailed t-tests were also conducted in R to identify differences between the ages within each sex. Samples were run in duplicates and the mean of individual replicates were plotted in scatterplot graphs with mean per group and standard error bars also depicted for each significant protein. In all analyses, the p-values were adjusted using false discovery rate (FDR), with significance being defined as FDR < 0.05. Log_2_(Fold Change) was calculated separately using LC/MS data by adding 1 to every value (Protein Abundance + 1), taking the average of the groups and then dividing the appropriate groups for each comparison followed by log_2_ transformation of the resulting values. All LC/MS statistical data and fold change values can be found in Additional file 2.

## Results

### Identification of developmentally regulated K48-polyubiquitin protein targets in the amygdala of 4- and 9-week-old animals

**Fig. 1:**
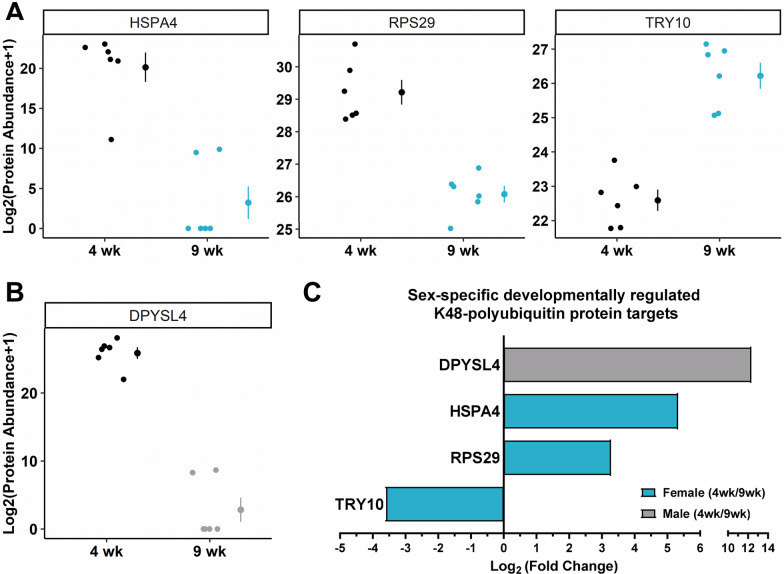
Sex-selective changes in K48-polyubiquitin protein targets in the amygdala of male and female rats across development. The basolateral amygdala (BLA) of 4-week-old (4 wk) and 9-week-old (9 wk) male and female rats was collected (*n* = 10 animals per group per sex per age). A subset of the samples was used for liquid chromatography mass spectrometry (LC/MS) to identify K48-polyubiquitin protein targets in whole cell protein lysates purified with a K48-specific tandem ubiquitin binding entity (K48-TUBE) (*n* = 6 animals per group per sex per age). The log_2_(Protein Abundance + 1) values are plotted for proteins identified as being targeted differently by K48-polyubiquitination across development in females (**A**) and males (**B**). **C** List of the proteins in both sexes that had increased or decreased levels in K48-polyubiquitin-purified samples across development in females (blue) and males (gray) with their corresponding Log_2_(Fold Change) at 4 wk compared to 9 wk. Data were a sum of two technical replicates. Log_2_(Fold Change) was calculated by adding 1 to every value (Protein Abundance + 1), taking the average of the groups and then dividing the appropriate groups for each comparison followed by log_2_ transformation of the resulting values

We previously reported adult female rats had higher baseline levels of K48-polyubiquitination in the amygdala compared to adult male rats, which was associated with increases in the transcriptional activator DNA 5-hydroxymethylcytosine mark at the promoter of one of the four ubiquitin coding genes, *Uba52,* and correlated with increased K48-polyubiquitination levels [18]. In order to better understand baseline differences in K48-polyubiquitin targeting of protein substrates and determine if the baseline sex differences observed in adults are developmentally regulated, we first isolated proteins marked by K48-polyubiquitination in the amygdala of 4-week-old (4 wk) and 9-week-old (9 wk), time points corresponding to pre- and post-puberty, male and female rats using a K48-specific tandem ubiquitin binding entity (TUBE) with LC/MS, which we have previously used to determine sex differences in K48-polyubiquitination targets during memory formation [19]. To determine if K48-polyubiquitin signaling changes across development within each sex, we first compared 4 wk and 9 wk female or male rats (Fig. 1). In total, we identified three proteins in female (Fig. 1A) and one protein in male rats (Fig. 1B) with changes in K48-polyubiquitin targeting. The one significant protein in males, dihydropyrimidinase-related protein 4 (DPYSL4; FDR_t-test_ = 0.0040), and two of the significant proteins in females, 40S ribosomal protein S29 (RPS29; FDR_t-test_ = 0.0240) and Heat shock 70-kDa protein 4 (HSPA4; FDR_t-test_ = 0.0240), had reduced targeting by K48-polyubiquitination at 9 wk. The third significant protein in female rats, Trypsin 10 (TRY10; FDR_t-test_ = 0.0181), had increased targeting by K48-polyubiquitination at 9 wk. The log_2_-fold changes in K48-polyubiquitin targeting at 4 wk compared to 9 wk were also calculated (Fig. 1C). Male rats had the largest log_2_-fold change (12.3) in K48-polyubiquitin targeting of DPYSL4. Conversely, the largest log_2_-fold change (5.3) difference in K48-polyubiquitin targeting in female rats occurred at HSPA4 and was less than half the magnitude observed in males, suggesting that although only one protein was identified in males, the difference in K48-polyubiquitin targeting across development in male rats occurs to a larger degree than in female rats. Though the number of differentially ubiquitinated proteins identified in our dataset is modest, together, these data indicate that K48-polyubiquitin protein targets change during development in both sexes.

**Fig. 2:**
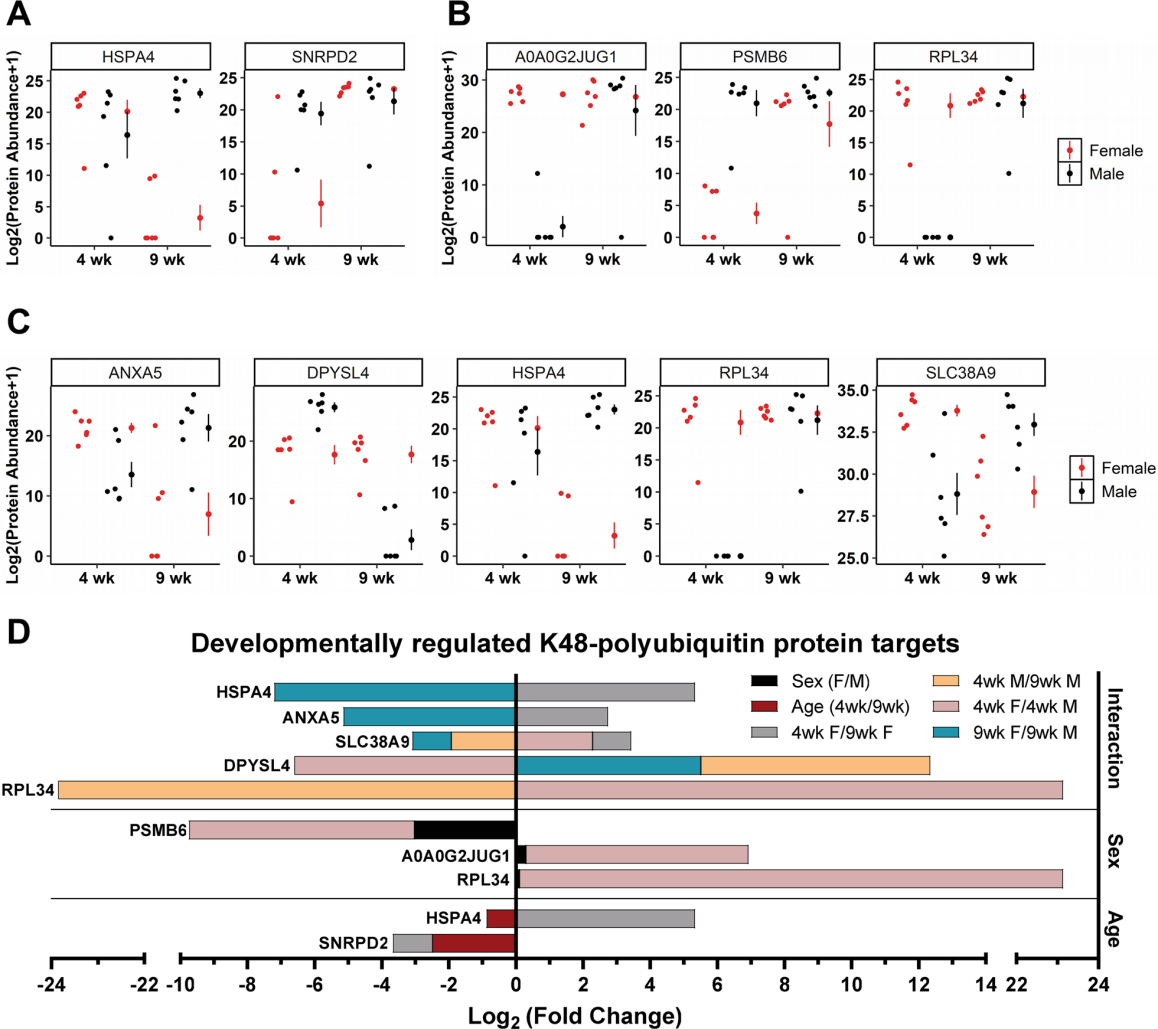
Identification of protein targets of K48-polyubiquitin in the amygdala of both sexes which differ as a result of sex, time, or both. The basolateral amygdala (BLA) of 4-week-old (4 wk) and 9-week-old (9 wk) male and female rats was collected from the same animals as Fig. 1. The same subset of animals was used for liquid chromatography mass spectrometry (LC/MS) to identify K48-polyubiquitin protein targets in whole cell protein lysates purified with a K48-specific tandem ubiquitin binding entity (K48-TUBE) (*n* = 6 animals per group per sex per age). The log_2_(Protein Abundance + 1) values are plotted for proteins identified as being targeted differently by K48-polyubiquitination as a result of Age (**A**), Sex (**B**), or and interaction of Sex X Age (**C**). **D** List of the proteins with corresponding Log_2_ (Fold Change) in both sexes that had increased or decreased levels in K48-polyubiquitin-purified due to Age (red) or Sex (black). For each protein, the corresponding Log_2_(Fold Change) is provided for significant differences in 4 wk vs 9 wk females (gray) or males (orange) or female vs male at 4 wk (pink) or 9 wk (blue) as indicated by Tukey’s post hoc analysis. Data were a sum of two technical replicates and Tukey’s honest significance difference post hoc test was used. Log_2_(Fold Change) was calculated by adding 1 to every value (Protein Abundance + 1), taking the average of the groups and then dividing the appropriate groups for each comparison followed by log_2_ transformation of the resulting values

After establishing the changes in K48-polyubiquitin targets within each sex across development, we next identified proteins that were differentially targeted by K48-polyubiquitination across Age, Sex, or as an interaction of Age X Sex (Fig. 2). First, we discovered two proteins with differences in K48-polyubiquitination targeting as a result of Age regardless of Sex (Fig. 2A). Small nuclear ribonucleoprotein Sm D2 (SNRPD2; FDR_glmm_ = 0.0166) had increased targeting by K48-polyubiquitination at 9 wk compared to 4 wk animals and post hoc analysis revealed increased targeting in 9 wk compared to 4 wk female rats (*p* < 0.0001), but no differences were observed in male rats (*p* = 0.9369). Heat shock 70 kDa protein 4 (HSPA4; FDR_glmm_ = 0.0175), which was also significant for an interaction between Age X Sex, was the second protein and had decreased targeting by K48-polyubiquitination in 9 wk compared to 4 wk animals. Post hoc analysis revealed increased targeting in 4 wk compared to 9 wk females (*p* < 0.0001), but decreased targeting in 9 wk female compared to male rats (*p* < 0.0001). Next, we discovered three proteins with differences in K48-polyubiquitination targeting as a result of Sex (Fig. 2B). IF rod domain-containing protein (A0A0G2JUG1; FDR_glmm_ = 0.0007) had less targeting by K48-polyubiquitination in male compared to female rats, while proteasome subunit beta type-6 (PSMB6; FDR_glmm_ = 0.0053) had less targeting by K48-polyubiquitination in female compared to male rats. Post hoc analysis revealed targeting differences for both proteins in 4 wk male vs female rats (p =  < 0.0001 for both proteins), but not 9 wk male vs female rats (A0A0G2JUG1 *p* = 0.9050; PSMB6 *p* = 0.4070). The third protein, 60S ribosomal protein L34 (RPL34; FDR_glmm_ < 0.0001), is targeted by K48-polyubiquitination more in female rats compared to male rats and also had a significant interaction of Age X Sex. Post hoc analysis revealed increased targeting by K48-polyubiquitination in 4 wk females compared to males (*p* < 0.0001) and in 9 wk males compared to 4 wk males (*p* < 0.0001). Lastly, we identified proteins that were not significantly different for Age or Sex but did have an interaction between Age X Sex, indicating K48-polyubiquitination targeting was either changing over time in one or both sexes. Five proteins had a significant interaction between Age X Sex (Fig. 2C), two of which were already mentioned above, RPL34 and HSPA4. Of the remaining three, K48-polyubiquitination targeting of Annexin A5 (ANXA5; FDR_glmm_ = 0.0223) and Sodium-coupled neutral amino acid transporter 9 (SLC38A9; FDR_glmm_ = 0.0104) changed following a similar pattern: Female rats had more targeting of both proteins at 4 wk compared to 9 wk (ANXA5 p = 0.0002; SLC38A9 p = 0.0006), but 9 wk male rats had more targeting of both proteins compared to 9 wk female rats (ANXA5 *p* = 0.0001; SLC38A9 *p* = 0.0068). However, SLC38A9 also had significant differences in K48-polyubiquitin targeting in 4 wk female compared to male rats (p = 0.0003) and in 4 wk vs 9 wk male rats (*p* = 0.0047). The fifth protein identified in our dataset as having a significant interaction between Age X Sex was dihydropyrimidinase-related protein 4 (DPYSL4; FDR_glmm_ = 0.0003) which was identified initially as changing across age in male rats. K48-polyubiquitination targeting of DPYSL4 was unchanged in female rats across development (*p* = 0.9999), but males had increased targeting compared to female rats at 4 wk (p = 0.0013) and decreased targeting compared to female rats at 9 wk (*p* < 0.0001). Additionally, 4 wk male rats had increased targeting compared to 9 wk male rats (*p* < 0.0001). Lastly, we calculated the log_2_-fold change of K48-polyubiquitin targeting of 4 wk compared to 9 wk animals for Age comparison and female compared to male for Sex comparison, as well as individual comparisons between 4 wk vs 9 wk females or males and 4wk or 9 wk females vs males (Fig. 2D). The log_2_-fold change is only shown for comparisons that were significant in the post hoc analysis. These fold change values indicate that RPL34 has the largest log_2_-fold change difference (-23.8 in 4 wk vs 9 wk male comparison and 23.1 in 4 wk female vs male comparison) in K48-polyubiquitin targeting of any protein identified, as this protein was not targeted by K48-polyubiquitination in 4 wk old male rats. Together these data demonstrate that Age and Sex alone or in combination can influence K48-polyubiquitination protein targets in the amygdala, indicating that sex-differences in the protein targets of K48-polyubiquitination are already present in the amygdala of 4-week-old rats and further change as a result of development.

### DNA methylation at the *Uba52* promoter and K48-polyubiquitination levels are developmentally regulated in female rats

**Fig. 3:**
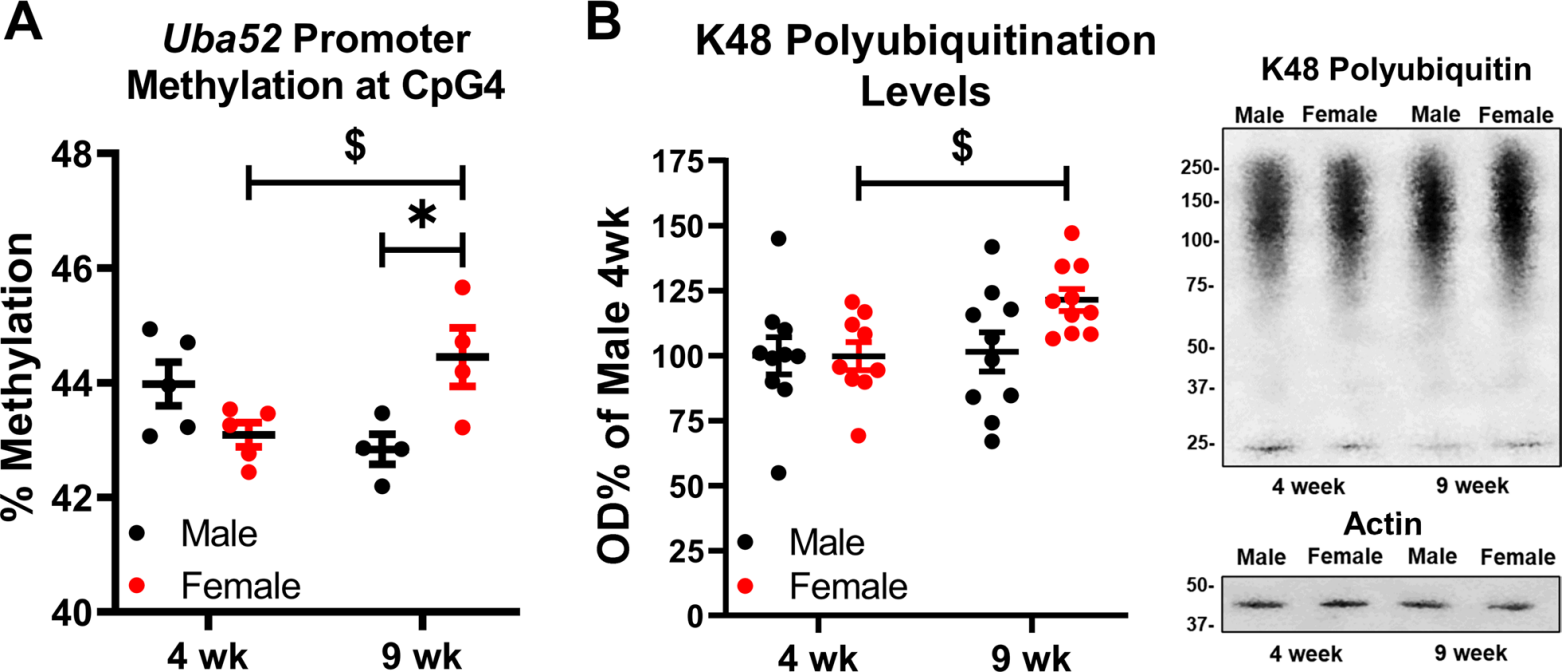
Methylation of the *Uba52* promoter and total K48 polyubiquitination levels are developmentally regulated within the amygdala of female rats. The second hemisphere of the basolateral amygdala (BLA) collected from the same animals used in Figs. 1 and 2 was used. Only a subset of the samples was used for the DNA methylation analyses. **A** Bisulfite sequencing was conducted to quantify methylation levels at CpG 4 of the *Uba52* promoter. T-test analysis (indicated by $ symbol) within each sex was significant for female rats, with 9 wk females having increased methylation levels compared to 4 wk females (two-tailed *t*-test, *t*_7_ = 2.664, *p* = 0.0323), but was not significant in male rats (two-tailed *t*-test, *t*_7_ = 2.353, *p* = 0.0509). Group sizes are as follows: *n* = 5 in 4 wk male and female, *n* = 4 in 9 wk male and female. Two-way ANOVA (indicated by * symbol) was not significant for Age (*F*_1,14_ = 0.0936, *p* = 0.7642) or Sex (*F*_1,14_ = 1.061, *p* = 0.3204), but there was an interaction between Sex X Age (*F*_1,14_ = 12.61, *p* = 0.0032). Tukey’s honest significance difference post hoc test determined 9 wk females had higher methylation levels than 9 wk males (*p* = 0.0368). Group sizes are as follows: *n* = 5 in 4 wk male and female, *n* = 4 in 9 wk male and female. **B** Total K48-polyubiquitination levels were quantified using western blot. Two-tailed t-test (indicated by $ symbol) of males revealed no differences between 4 and 9 wk animals (two-tailed *t*-test, *t*_18_ = 0.1400, *p* = 0.8902), but 9 wk females had significantly higher K48-polyubiquitination levels compared to 4 wk females (two-tailed *t*-test, *t*_17_ = 3.190, *p* = 0.0054). Two-way ANOVA did not show significant differences for Age (*F*_1,35_ = 3.379, *p* = 0.0745), Sex (*F*_1,35_ = 2.457, *p* = 0.1260), or interaction between Sex X Age (F_1,35_ = 2.581, p = 0.1172). In representative western blot images, the top box is K48 polyubiquitination and the bottom box is β-actin, which was used as a loading control. Group sizes are as follows: *n* = 10 in 4 wk male, 9 wk male, and 9 wk female, *n* = 9 in 4 wk female. OD values were normalized to 4 wk males. *p < 0.05 in ANOVA and ^$^*p* < 0.05 in *t*-test

We next wanted to determine the relationship between changes in K48-polyubiquitin signaling and the molecular mechanisms regulating total ubiquitin levels across development. As mentioned above, we previously observed increased methylation at CpG 4 in the *Uba52* promoter at baseline, which correlated with increased K48-polyubiquitin levels in the amygdala of female compared to male rats at 9 wk. To determine whether these molecular differences seen in the amygdala of 9-week-old adult animals are developmentally regulated, we first examined DNA methylation levels at the *Uba52* promoter in the amygdala of the same 4 wk and 9 wk male and female rats used for our proteomic analysis. Using direct bisulfite sequencing, we determined patterns of DNA methylation at the *Uba52* promoter in our rats. We first wanted to determine if changes in percentage of methylation were occurring within each sex across development before looking at Sex and Age as independent variables. Two-tailed t-test analyses revealed a significant increase in methylation levels in 9 wk female rats compared to 4 wk at the *Uba52* promoter at CpG 4 (Fig. 3A). There were no differences in CpG 1, 2, 3, or overall methylation levels within each sex (Additional file 1: Fig. S1), indicating that female, but not male, rats seem to have changes in methylation levels only at CpG 4 across development.

After establishing baseline changes in methylation status, we used two-way ANOVA to compare Age and Sex independently. At CpG 4 of the *Uba52* promoter, there was an interaction between Age X Sex where male and female rats do not differ in methylation levels at 4 wk, but at 9 wk female rats have more methylation than 9 wk male rats at this CpG site (Fig. 3A). In addition to CpG 4, we looked at methylation of other CpG sites in the *Uba52* promoter including 1, 2, and 3 and overall methylation of CpG 1–4 was calculated (Additional file 1: Fig. S2). There were no changes in CpG 1 and 3 or overall methylation. However, there was a main effect for Sex, but not Age, at CpG 2, meaning male rats having higher methylation levels than female rats at CpG 2 independent of Age, but within each Age, levels are not different between the sexes (Additional file 1: Fig. S2B). Together, these data are in agreement with our previous findings in 9-week-old adult male and female rats where we observed increased methylation at CpG 4 of the *Uba52* promoter in the amygdala of females compared to males. Importantly, our present data reveal that these sex differences are developmentally regulated as methylation at CpG 4 of the *Uba52* promoter did not differ between 4 wk male and female rats.

Next, we quantified overall K48-polyubiquitination levels in the amygdala of our 4 wk and 9 wk rats to determine if epigenetic regulation of *Uba52* by DNA methylation correlates to overall protein level and if the increased baseline levels of K48-polyubiquitination previously observed in the amygdala of 9-week-old adult female rats compared to males was developmentally regulated. Again, we first ran t-test analyses to determine if changes in K48-polyubiquitination levels were occurring within each sex across development before looking at Sex and Age as independent variables. We found no differences between 4 wk male compared to 9 wk male rats, but we observed increased K48-polyubiquitination levels in 9 wk female compared to 4 wk female rats (Fig. 3B), indicating that methylation at CpG 4 of the *Uba52* promoter as well as protein level are increased across development in female rats, but neither change across development in male rats. After conducting a two-way ANOVA to compare Age and Sex independently, we observed a trend for a main effect for Age, but no main effect for Sex and no interaction between Age X Sex, indicating that changes in methylation of CpG 4 on the *Uba52* promoter did not translate to a statistically reliable effect for either variable or an interaction of the variables at the protein level (Fig. 3B). Due to puberty-dependent changes in hippocampus activity during learning being reported in mice [28], we also quantified K48-polyubiquitination levels in the hippocampus, but we observed no changes between groups (Additional file 1: Fig. S3). Together with our proteomic analyses, these data indicate that methylation levels at CpG 4 of the *Uba52* promoter and protein-specific K48 polyubiquitination change as a result of an interaction between age and sex, but global K48 polyubiquitination levels do not.

### Adult female, but not male, rats require K48-polyubiquitin signaling in the amygdala for context fear memory formation

**Fig. 4:**
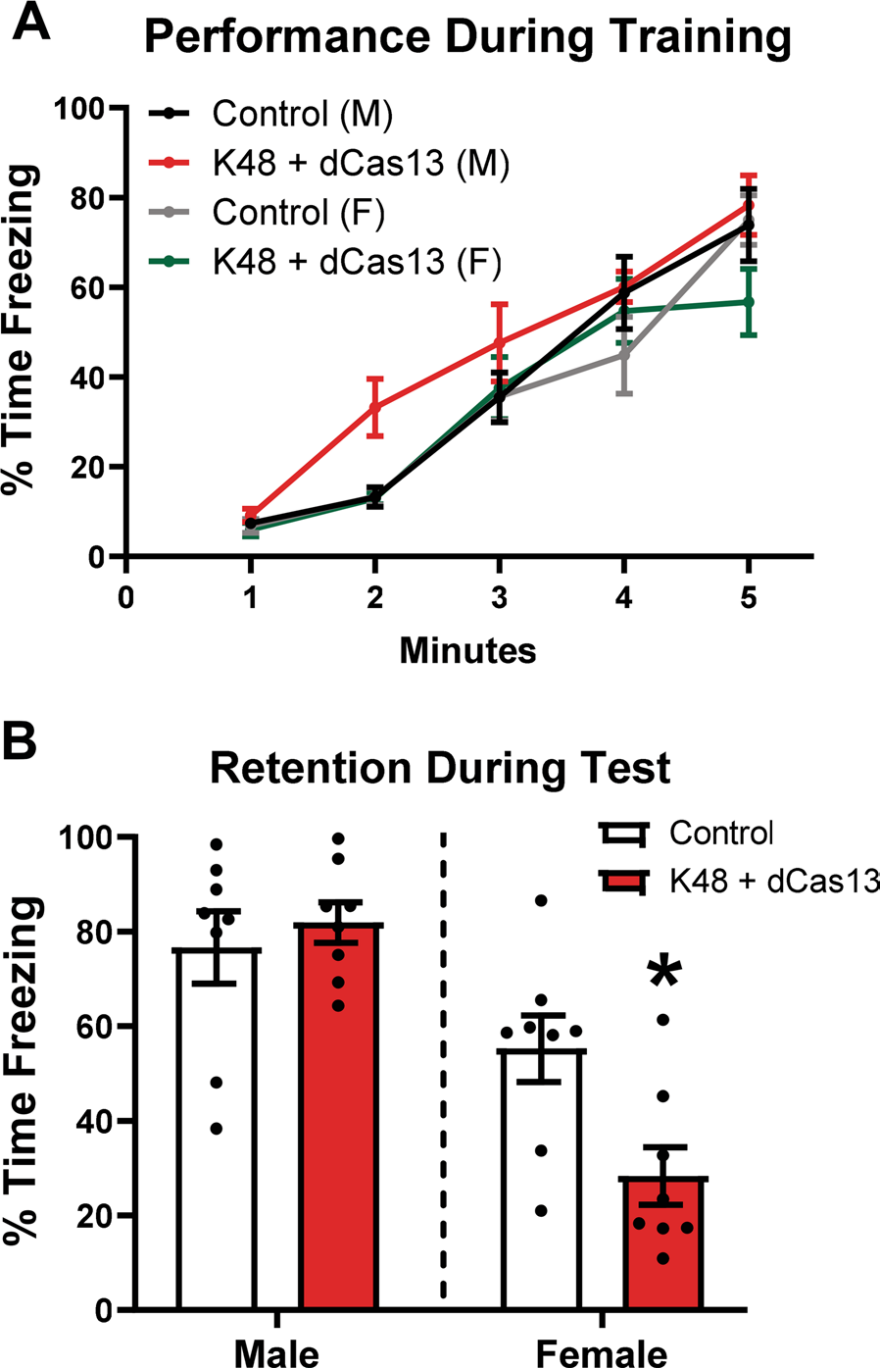
CRISPR-dCas13b-ADAR2DD mediated knockdown of K48-polyubiquitination in the BLA impairs contextual fear memory in female, but not male, rats. Young adult male and female rats (*n* = 8 per group per sex) received a bilateral infusion of either gRNA targeting K48 and dCas13b-ADAR2DD plasmids (K48 + dCas13; Treatment) or dCas13b-ADAR2DD plasmid alone (Control) into the BLA. Twenty-eight days later, rats were trained to contextual fear conditioning and tested the following day. **A** During contextual fear conditioning, we found a main effect for Time in (*F*_2.95,82.61_ = 91.21, *p* < 0.0001) and Sex (*F*_1,28_ = 6.572, *p* = 0.0160), but there was no main effect for Treatment (*F*_1,28_ = 1.242, *p* = 0.2746), nor was there a significant interaction of Time X Sex (F_4,112_ = 0.4820, *p* = 0.7489), Time X Treatment (*F*_4,112_ = 1.464, *p* = 0.2178), Sex X Treatment (*F*_1,28_ = 2.742, *p* = 0.1089), or Time X Sex X Treatment (F_4,112_ = 1.412, *p* = 0.2346). **B** During testing, there was no main effect for Treatment (*F*_1,28_ = 2.866, *p* = 0.1016), but there was a main effect for Sex (*F*_1,28_ = 34.40, *p* < 0.0001) and there was a significant interaction of Treatment X Sex (*F*_1,28_ = 6.358, *p* = 0.0177). Bonferroni post hoc analysis determined that K48 + dCas13 males had no difference in performance compared to control males (*p* > 0.9999), but K48 + dCas13 females had significantly impaired memory compared to control females (*p* = 0.0118), indicating that the K48-polyubiquitin knockdown only impaired memory retention in females. *p < 0.05

After determining sex differences in K48-polyubiquitin regulation, abundance, and protein targets developmentally regulated, we wondered whether the increased prevalence of PTSD in women compared to men could be related to sex-differences in the role of K48-polyubiquitin signaling within the amygdala during fear memory formation. To determine if K48-polyubiquitination was necessary in the amygdala of male and female rats for fear memory formation, we used the CRISPR-dCas13b-ADAR2DD system to alter the sequence coding for K48 on the transcripts of the ubiquitin gene coding for the largest number of total ubiquitin proteins, *Ubc*, as *Uba52* only encodes for a single ubiquitin molecule. The CRISPR-dCas13b-ADAR2DD system essentially generates a K48 knockdown without altering the abundance of the other ubiquitin linkage sites, allowing us to isolate the role of K48-polyubiquitination in the amygdala during fear memory formation. The guide RNA (gRNA) was developed and validated in our prior study, where we used the same system to target the K63 linkage site of ubiquitin during fear memory formation [20]. We injected either K48 gRNA with the dCas13b-ADAR2DD plasmid (K48 + dCas13) or dCas13b-ADAR2DD plasmid alone (Control) into the BLA of 9 wk male and female rats and then waited 28 days to allow for the plasmids to express and then RNA to be edited and translated into altered ubiquitin protein. Due to the timeline from surgery to training (4 weeks), we chose to use only 9-week-old adult rats rather than also including prepubescent rats as the plasmids would not reach optimal expression levels until after puberty if injected at younger ages. After 28 days, animals were trained to contextual fear conditioning and then tested one day later for memory retention. During training, we observed a main effect for Sex and Time, but not Treatment. We also did not observe any interactions, indicating that although male and female rats performed differently during training, the K48 knockdown did not impact fear learning in either sex (Fig. 4A). During testing, we did not observe a main effect for Treatment, but there was a main effect for Sex and an interaction between Sex X Treatment (Fig. 4B). Due to this interaction, we used Tukey’s post hoc analysis to compare Control vs. Treatment in each sex and determined that K48 + dCas13 female rats had significantly impaired memory compared to control female rats. However, memory retention in males did not differ between groups. Together, these data suggest that female, but not male, rats require K48-polyubiquitin signaling in the amygdala for fear memory formation, implicating K48-polyubiquitination as a potential target for better understanding molecular mechanisms underlying the disparity in PTSD development between the sexes.

## Discussion

There are various reports of sex-differences in K48-polyubiquitination both at baseline and in the process of memory formation [15, 18, 19]. We previously observed increased baseline levels of K48-polyubiquitination in the amygdala of 9-week-old adult female rats compared to males [18], as well as different protein targets during fear memory formation [19]. However, it is unclear when, how, and why these sex-differences occur. Here, we identified developmentally regulated changes in methylation at the *Uba52* promoter and K48-polyubiquitination levels in the amygdala of female rats as well as K48-polyubiquitination protein targets in the amygdala of male and female rats. Additionally, we discovered that 9-week-old adult female, but not male, rats require K48-polyubiquitination in the amygdala for fear memory formation. Together, these data are the first to determine a timeline for changes in baseline K48-polyubiquitination levels and targets in the amygdala, with changes in DNA methylation as a potential driver, and identify a sex-selective role for K48-polyubiquitination in the amygdala of 9-week-old adult animals for fear memory formation.

Our prior study examining sex differences in K48-polyubiquitination during memory consolidation in the amygdala did not identify the protein targets of K48-polyubiquitination at baseline, nor did it examine if these differences between males and females were already present earlier in development [19]. In the current study, LC/MS was conducted to identify protein targets of K48-polyubiquitination in the amygdala at baseline of male and female rats that were either 4 wk or 9 wk of age. Our proteomic analysis revealed differences in K48-polyubiquitin targeting within each sex across development as we found one and three significant proteins in male and female rats, respectively. The protein identified in male rats was DPYSL4, which has been shown to have persistent expression in the adult rat hippocampus [38] and regulate dendrite morphology in mice [39]. Additionally, a prior study also observed decreased DPYSL4 abundance in adult rat brains compared to neonatal brains [40], with no sex differences being observed. However, it is unclear what brain region was examined during that study. Together, these data seem to indicate that targeting of DPYSL4 by K48-polyubiquitination is unchanged in the female rat amygdala across development but is decreased in the male rat amygdala across development. The three proteins identified in female rats were TRY10, HSPA4, and RPS29. TRY10, which is known to be expressed in the brain [41], was the only protein of the three identified which had a higher abundance in our K48-purified amygdala samples in 9 wk compared to 4 wk female rats. HSPA4 facilitates protein folding as well as targeted degradation of misfolded proteins; one study found that the amygdala of aged females has a higher abundance of HSPA4 compared to the amygdala of aged males in human tissue [42]. Together with the current findings, it is possible that HSPA4 may be targeted by K48-polyubiquitination for degradation to a larger degree in the amygdala of adult male rats than in adult female rats due to differences in total HSPA4 levels. RPL34 was also identified and has been reported to have higher expression in the medial prefrontal cortex (mPFC) of male compared to female rats [43]. The same study also reports a sex difference in *Uba52* expression, where they found higher expression in the mPFC of male compared to female rats regardless of stress, which is opposite of what we previously observed in the amygdala [18]. Regardless, RPL34 is involved in the regulation of neural cell growth and proliferation and our data suggest that RPL34 may be more important for amygdala development in young (4-week-old) male, compared to female, rats. Future studies should investigate sex differences in the levels and roles of the proteins identified in the current study within the amygdala across development.

In our previous study, we observed sex differences in methylation levels of the *Uba52* promoter and K48-polyubiquitination levels in the amygdala of 9-week-old adult rats [18]. Here, we extend those findings by revealing that these differences are developmentally regulated, where there are increases in female rats across age but not in male rats. These data suggest that K48-polyubiquitin signaling increases in the amygdala of female, but not male, rats as a consequence of age, which suggests an importance of sexual maturity in females on K48-polyubiquitin signaling in young adulthood. While not directly tested here, it would be interesting to speculate that prevention of sexual maturity via ovariectomy prior to puberty might normalize the sex differences in K48-polyubiquitin signaling in adulthood. Future studies should include ovariectomized females and castrated males into the experimental design and measure hormone levels at time of brain collection to gain a better understanding of the contribution of sex maturity and sex hormones to differences in K48-polyubiquitin signaling in young adulthood.

In humans, there is evidence of a large deceleration of amygdala volume expansion in females during puberty [25, 44] and a hypothesis for a mechanism of estrogenic regulation of ubiquitin signaling has been proposed [45]. Considering this, it is possible that molecular mechanisms in the amygdala may be changing due to hormonal signaling during a time period of slowed volume expansion in females, which could explain our findings. However, it is important to consider that our animals were supplied by an outside vendor and shipping induces stress in the animals, which could have impacted our findings. Studies in rats have shown that early life stress has sex-specific effects on connectivity strength between the amygdala and other brain regions [46] as well as miRNA expression [47] and DNA methylation [48] levels in the amygdala. Due to this, we cannot rule out the potential effect of stress from the shipping procedure on K48-polyubiquitination activity in the amygdala. Future studies will be conducted using in-house animal colonies to avoid exposing animals to early life stress.

We previously used the CRISPR/dCas9 system to up or downregulate genes coding for ubiquitin and the proteasome complex and found that only male rats had increased global K48-polyubiquitination levels after fear learning, but both sexes required protein degradation in the amygdala for fear memory formation [18]. Here, we used the CRISPR-dCas13b-ADAR2DD system to specifically knockdown the K48 linkage in the amygdala of male and female rats instead of targeting overall ubiquitin levels. This approach revealed a sex-selective necessity for K48-polyubiquitination in the amygdala of female, but not male, rats for fear memory formation. Interestingly, using the CRISPR-dCas13b-ADAR2DD system, we recently identified a sex-selective role for the proteasome-independent lysine-63 (K63) polyubiquitin mark during fear memory formation in the amygdala, as female, but not male, rats required K63-polyubiquitination for fear memory formation and no proteins in the male rat amygdala gained the mark following fear conditioning [20]. In another study, we utilized the same K48-specific TUBE used here and identified sex-specific protein targets of K48-polyubiquitination in the amygdala of rats during fear memory formation [19]. It is peculiar that we identified increased K48-polyubiquitination levels and differential protein targets following fear learning in the amygdala of male rats in our previous studies, but our current study indicates that K48-polyubiquitination is not necessary for fear memory formation in the amygdala of male rats. This could be because the K48 mark is simply not necessary for fear memory formation in male rats, though this seems unlikely considering the broad role for protein degradation in memory formation. However, the loss of the K48 linkage in the amygdala of males could be compensated for by another polyubiquitin linkage or we can speculate that female rats require K48-polyubiquitination in the amygdala for fear memory formation, while male rats require other polyubiquitin marks. Furthermore, our male animals had high average freezing percentages, which could suggest a potential ceiling effect. Thus, it is possible that K48 knockdown could have enhanced memory in males but not have been detected beyond the high level of freezing currently observed. Future studies should use weaker training protocols in males to prevent a potential ceiling effect during behavioral testing, as well as focus on identifying all polyubiquitin linkages necessary for memory formation in the amygdala and other brain regions of both sexes to identify sex differences and potential therapeutic targets.

Importantly, while the current findings provide insight into sex differences related to developmental baseline regulation and memory-related necessity of K48-polyubiquitin signaling in the amygdala, but much still remains unknown and there are limitations of this study that must be considered. Firstly, our study lacks whole proteome data to compare our K48-purified proteome against, which could have been used to normalize the K48-purified protein targets to determine true changes in K48-polyubiquitination. The current interpretation relies on the assumption that an increased abundance in K48-purified samples indicates increased targeting of that particular protein by K48-polyubiquitination, and vice-versa for decreased abundance. Regardless, it is not surprising that proteins targeted for degradation by K48-polyubiquitination would change over time as the brain grows. We were also unable to determine the role of K48-polyubiquitination in the amygdala of prepubescent animals due to the timeline of the CRISPR-dCas13b-ADAR2DD experiment which required long incubation periods to get optimal plasmid expression. It would be beneficial to determine if the *necessity* of the K48-polyubiquitin mark in fear memory formation is developmentally regulated, though technical limitations make this especially challenging to test. Lastly, the minimal timepoints for data collection are a limitation of the current study. In order to better understand the role of K48-polyubiquitin signaling in the amygdala between the sexes, future studies should include multiple timepoints such as neonates and aged animals. 

## Perspectives and significance

Our results support the need for further studies into sex-differences in the brain, particularly in abundant molecular mechanisms such as the UPS. There are numerous studies that report sex-differences in the brain at baseline and during memory formation, with sexual maturity playing a role. However, our study highlights the importance of determining sex-differences at the molecular level in each sex to better understand how diseases with sex-differences in prevalence occur and for the eventual development of sex-appropriate treatments. Moreover, our results challenge the long-held belief that K48-polyubiquitination was the major degradation mark in the amygdala for fear memory formation, at least in male rats, suggesting that further investigation is needed on the role of each polyubiquitin linkage in memory formation and how this varies between sexes.

## Conclusions

In conclusion, we present a detailed analysis of baseline and memory-related sex differences in K48-polyubiquitin signaling in the amygdala across development. We identified protein targets of K48-polyubiquitination in the amygdala before and after sexual maturity and observed developmentally driven changes in methylation at the *Uba52* promoter and overall K48-polyubiquitination levels in the amygdala of female, but not male, rats. Lastly, we determine that female, but not male, rats require K48-polyubiquitin signaling in the amygdala for fear memory formation. Together, these data provide a better understanding of K48-polyubiquitin signaling in each sex across development and for fear memory, which is necessary for addressing sex bias in research to better serve both sexes. With this information, we can better understand sex differences in mechanisms that involve K48-polyubiquitin signaling, such as PTSD, synaptic plasticity and memory formation.

### Supplementary Information

**Additional file 1: Figure S1. **Methylation at CpG 1-3 and overall methylation of the Uba52 promoter are not developmentally regulated in the amygdala of either sex. **Figure S2.** Methylation at CpG 1-3 and overall methylation of the Uba52 promoter in the amygdala are not influenced by sex or age. **Figure S3. **K48-polyubiquitination levels in the hippocampus are not influenced by sex or age.

**Additional file 2. **Proteomics data.

## Data Availability

The datasets generated and/or analyzed during the current study are available in the ProteomeXchange Consortium via the PRIDE [1] partner repository with the dataset identifier PXD042021 and 10.6019/PXD042021.
