# Supplementary material for: Sex-differences in proteasome-dependent K48-polyubiquitin signaling in the amygdala are developmentally regulated in rats
Source: Biol Sex Differ. 2023 Nov 10;14:80. doi: 10.1186/s13293-023-00566-z (PMC10638793; doi:10.1186/s13293-023-00566-z)
Supplement: Supplementary file 1 — Additional file 1: Figure S1. Methylation at CpG 1-3 and overall methylation of the Uba52 promoter are not developmentally regulated in the amygdala of either sex. Figure S2. Methylation at CpG 1-3 and overall methylation of the Uba52 promoter in the amygdala are not influenced by sex or age. Figure S3. K48-polyubiquitination levels in the hippocampus are not influenced by sex or age. [file 13293_2023_566_MOESM1_ESM.pdf]

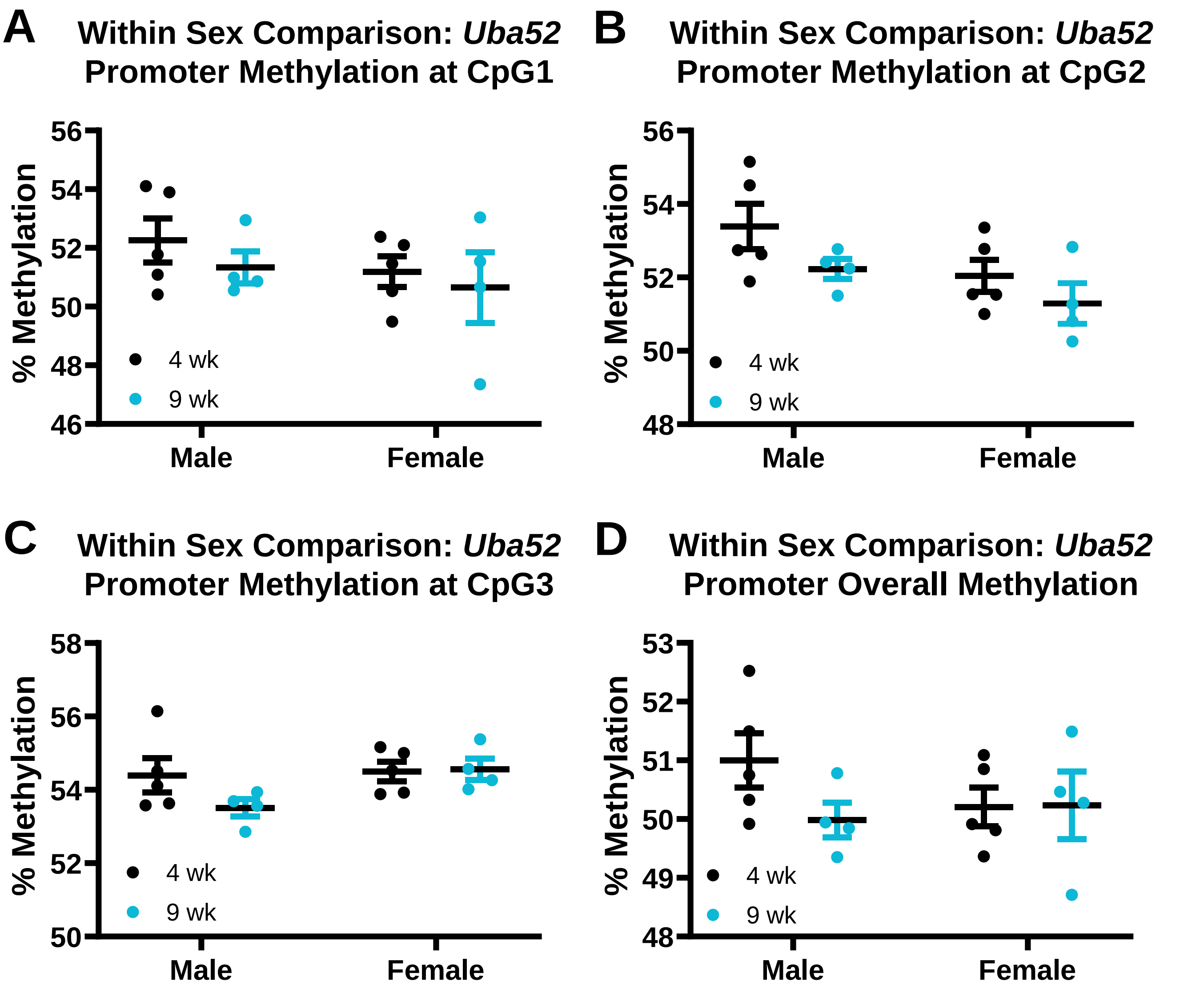

Supplemental Figure 1. Methylation at CpG 1-3 and overall methylation of the *Uba52* promoter are not developmentally regulated in the amygdala of either sex. The second hemisphere of the basolateral amygdala (BLA) collected from the same animals used in **Figure 1 and 2** was used. Only a subset of the samples was used for the DNA methylation analyses. Bisulfite sequencing was conducted to quantify methylation levels at CpG 1-3 and overall methylation (CpG 1-4) of the *Uba52* promoter. **(A)** There were no significant differences in methylation levels of the *Uba52* promoter at CpG 1 in males (two-tailed t-test, $t_7 = 0.9423$, $p = 0.3774$) or females (two-tailed t-test, $t_7 = 0.4471$, $p = 0.6683$) when comparing 4 wk and 9 wk animals. **(B)** There were no significant differences in methylation levels of the *Uba52* promoter at CpG 2 in males (two-tailed t-test, $t_7 = 1.563$, $p = 0.1621$) or females (two-tailed t-test, $t_7 = 1.075$, $p = 0.3180$) when comparing 4 wk and 9 wk animals. **(C)** There were no significant differences in methylation levels of the *Uba52* promoter at CpG 3 in males (two-tailed t-test, $t_7 = 1.546$, $p = 0.1661$) or females (two-tailed t-test, $t_7 = 0.1416$, $p = 0.8914$) when comparing 4 wk and 9 wk animals. **(D)** The overall methylation was calculated as the average methylation of CpG 1-4. There were no significant differences in methylation levels of the *Uba52* promoter at CpG 2 in males (two-tailed t-test, $t_7 = 1.750$, $p = 0.1236$) or females (two-tailed t-test, $t_7 = 0.0468$, $p = 0.9640$) when comparing 4 wk and 9 wk animals. Group sizes are as follows: n = 5 in 4 wk male and female, n = 4 in 9 wk male and female.

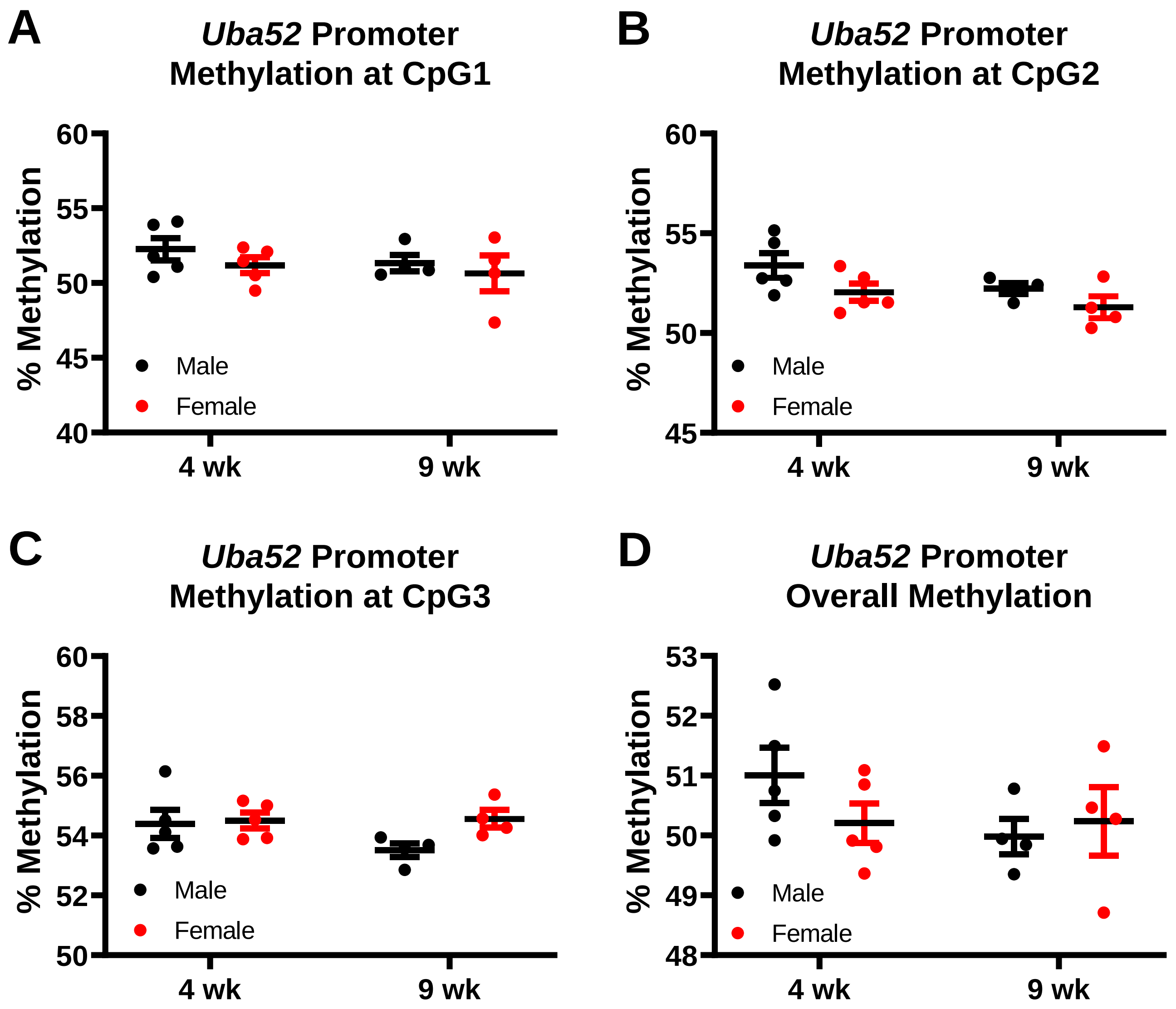

Supplemental Figure 2. Methylation at CpG 1-3 and overall methylation of the *Uba52* promoter in the amygdala are not influenced by sex or age. The second hemisphere of the basolateral amygdala (BLA) collected from the same animals used in **Figure 1 and 2** was used. Only a subset of the samples was used for the DNA methylation analyses. Bisulfite sequencing was conducted to quantify methylation levels at CpG 1-3 and overall methylation (CpG 1-4) of the *Uba52* promoter. **(A)** At CpG 1, there were no main effects for Age ($F_{1,14}$ = 0.8784, p = 0.3645) or Sex ($F_{1,14}$ = 1.270, p = 0.2787), nor was there an interaction between Sex X Age ($F_{1,14}$ = 0.0569, p = 0.8150). **(B)** At CpG 2, there was a main effect for Sex ($F_{1,14}$ = 5.078, p = 0.0408) and a trend for a main effect for Age ($F_{1,14}$ = 3.513, p = 0.0819). There was no interaction between Sex X Age ($F_{1,14}$ = 0.1587, p = 0.6964). **(C)** At CpG 3, there were no main effects for Age ($F_{1,14}$ = 1.411, p = 0.2547) or Sex ($F_{1,14}$ = 2.752, p = 0.1193), nor was there an interaction between Sex X Age ($F_{1,14}$ = 1.821, p = 0.1986). **(C)** The overall methylation was calculated as the average methylation of CpG 1-4. There were no main effects for Age ($F_{1,14}$ = 1.344, p = 0.2658) or Sex ($F_{1,14}$ = 0.3986, p = 0.5380), nor was there an interaction between Sex X Age ($F_{1,14}$ = 1.507, p = 0.2398). Group sizes are as follows: n = 5 in 4 wk male and female, n = 4 in 9 wk male and female.

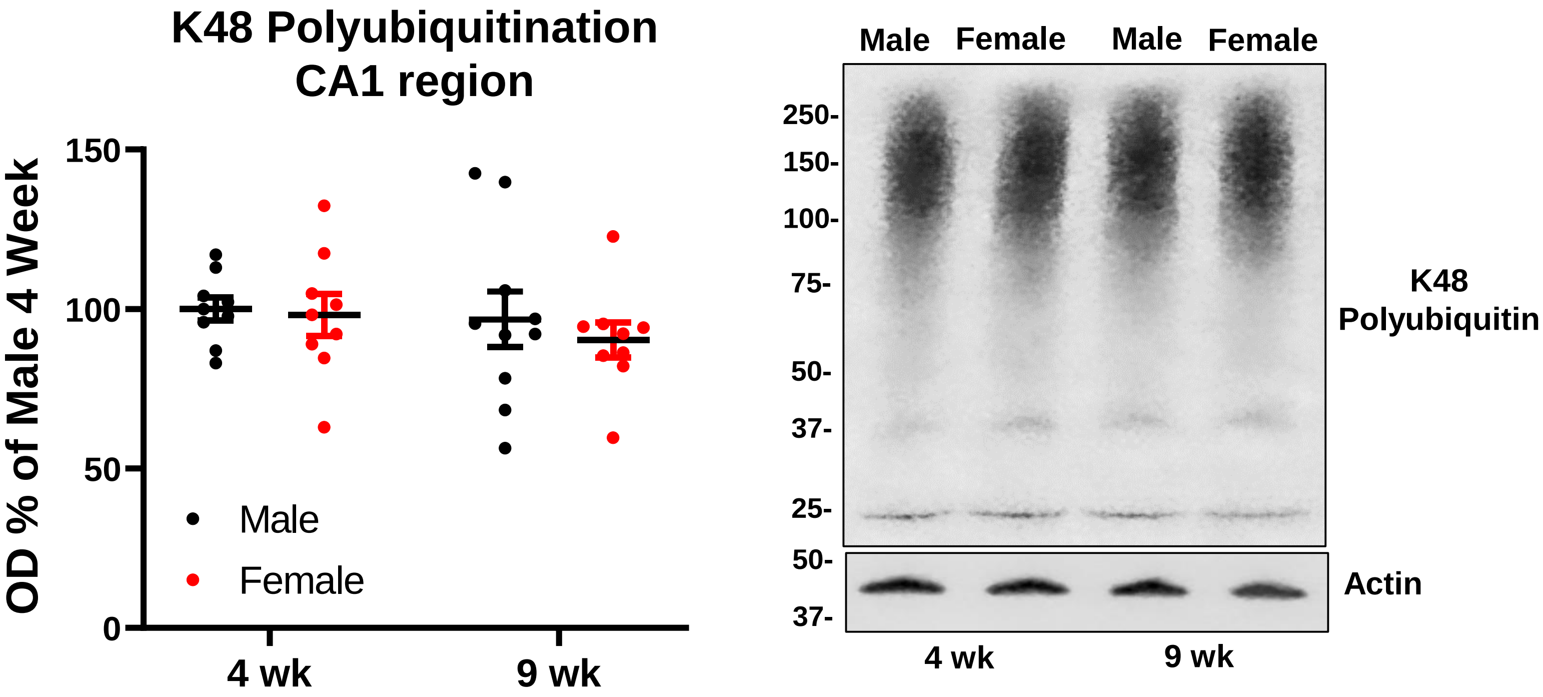

Supplemental Figure 3. K48-polyubiquitination levels in the hippocampus are not influenced by sex or age. The CA1 region of the hippocampus was collected from the same animals used in **Figure 1 and 2**. Whole cell lysates were collected and K48-polyubiquitination levels were quantified using western blot. There were no main effects for Age ($F_{1,33}$ = 0.7142, p = 0.4041) or Sex ($F_{1,33}$ = 0.4071, p = 0.5279), nor was there an interaction between Sex X Age ($F_{1,33}$ = 0.1203, p = 0.7309) for total K48-polyubiquitination levels in the CA1 region of the hippocampus. Group sizes are as follows: n = 9 in 4 wk male, 4 wk female, and 9 wk female, n = 10 in 9 wk male and male.
